# Lean-NET-Based Local Brain Connectome Analysis for Autism Spectrum Disorder Classification

**DOI:** 10.3390/bioengineering13010099

**Published:** 2026-01-15

**Authors:** Aoumria Chelef, Demet Yuksel Dal, Mahmut Ozturk, Mosab A. A. Yousif, Gokce Koc

**Affiliations:** 1 Department of Biomedical Engineering, Institute of Graduate Studies, Istanbul University-Cerrahpasa, Istanbul 34320, Türkiye; aoumria.chelef@ogr.iuc.edu.tr (A.C.); mosababoi@ogr.iuc.edu.tr (M.A.A.Y.); 2 Department of Electrical and Electronics Engineering, Engineering Faculty, Fatih Sultan Vakıf University, Istanbul 34015, Türkiye; dyukseldal@fsm.edu.tr; 3 Department of Electrical and Electronics Engineering, Engineering Faculty, Istanbul University-Cerrahpasa, Istanbul 34320, Türkiye; mahmutoz@iuc.edu.tr; 4 Department of Biomedical Engineering, Engineering and Architecture Faculty, Istanbul Yeni Yuzyil University, Istanbul 34010, Türkiye

**Keywords:** autism spectrum disorder ASD, rs-FMRI BOLD signal, graph learning, sparse functional brain connectome (Lean-NET), local graph metrics, feature selection, support vector machine (SVM)

## Abstract

Autism spectrum disorder (ASD) is a neurodevelopmental condition characterized by impairments in social interaction and communication, along with atypical behavioral patterns. Affected individuals often seem isolated in their inner world and exhibit particular sensory reactions. The World Health Organization has indicated a persistent increase in the global prevalence of autism, with approximately 1 in 127 persons affected worldwide. This study contributes to the growing research effort by presenting a comprehensive analysis of functional connectivity patterns for ASD prediction using rs-fMRI datasets. A novel approach was used for ASD identification using the ABIDE II dataset, based on functional networks derived from BOLD signals. The sparse functional brain connectome (Lean-NET) model is employed to construct subject-specific connectomes, from which local graph metrics are extracted to quantify regional network properties. Statistically significant features are selected using Welch’s *t*-test, then subjected to False Discovery Rate (FDR) correction and classified using a Support Vector Machine (SVM). Our experimental results demonstrate that locally derived graph metrics effectively discriminate ASD from typically developing (TD) subjects and achieve accuracy ranging from 70% up to 91%, highlighting the potential of graph learning approaches for functional connectivity analysis and ASD characterization.

## 1. Introduction

Autism spectrum disorders (ASD) are neurodevelopmental conditions that emerge early in childhood and persist into adulthood, characterized by social-communication difficulties and behavioral abnormalities [1]. Beyond behavioral definitions, neuroimaging studies suggest that ASD also involves atypical functional connectivity between brain regions; this view—often described as a functional disconnection syndrome—highlights disrupted communication across large-scale neural networks [2]. Accordingly, network-based resting-state fMRI (rs-fMRI) has become an important tool for studying ASD. Rs-fMRI is a non-invasive technique that measures blood-oxygen-level-dependent (BOLD) signals to characterize intrinsic brain activity [3]. In this context, the Autism Brain Imaging Data Exchange II (ABIDE II) provides a large multisite rs-fMRI resource for investigating connectivity alterations in ASD [4,5]. Although both single-site and multisite ABIDE data have been used, single-site cohorts are often preferred to reduce variability due to scanner and acquisition differences.

Several studies have leveraged ABIDE rs-fMRI for ASD–TD classification using diverse methodological pipelines. On single-site data (NYU), Thomas et al. [6] applied a 3D convolutional neural network (3D-CNN) to temporal BOLD representations, while Chu et al. [7] proposed a multi-scale graph representation learning framework to model hierarchical functional connectivity (FC). Other works derived time–frequency scalogram features via continuous wavelet transform (CWT) and evaluated multiple machine learning (ML) classifiers, reporting high accuracy in some settings [8].

Extending to multisite cohorts, a range of FC-based machine learning approaches has been reported, such as SVM/kSVM, logistic regression, and random forest, in addition to hybrid deep learning pipelines combining an FC matrix with CNN architectures [9,10,11,12,13]. Notably, performance has been shown to depend strongly on preprocessing and feature construction, and recent comparisons suggest that classical models—particularly (kernel) SVM—often remain competitive with or outperform several deep learning alternatives such as TabNet, XGBoost, and MLP on FC-derived features (AUC ≈ 0.75–0.77) [14,15].

To address the limitations of traditional FC and ML approaches, D.Y. Dal employed graph-theoretical metrics derived from multimodal (structural and functional) brain connectomes with the aim of identifying network signatures, achieving notable results [16]. A recent work, Yang et al. proposed a multi-view low-rank subspace graph structure learning method that integrates multi-atlas and multi-center rs-fMRI data to reduce heterogeneity and extract consistent FC features, achieving an accuracy of 83.2% on the ABIDE dataset [17].

Despite these advances, two methodological gaps remain salient. First, many pipelines still derive functional connectivity (FC) from dense pairwise Pearson/partial correlations between regional BOLD time series. While widely used, correlation-based FC can be sensitive to noise, preprocessing decisions, and indirect dependencies among regions, potentially inflating spurious edges and reducing subject-level reliability—an issue that becomes more pronounced when individual-level interpretation is required. Second, classification-driven studies often prioritize predictive performance without a rigorous, localized characterization of which regions drive group differences, including appropriate control for multiple comparisons when identifying discriminative regions. Consequently, stable and interpretable node-level signatures that generalize beyond global summary metrics remain comparatively underdeveloped in the ASD rs-fMRI literature.

Accordingly, there remains a fundamental methodological imperative to establish rigorous, data-driven graph construction methods that can infer subject-specific functional brain networks while mitigating noise, reducing spurious connections, and preserving neurobiological interpretability—especially in the context of heterogeneous multisite datasets. Addressing this need is essential for identifying stable and localized connectivity alterations associated with ASD, beyond global network summaries or purely performance-driven classification models. To improve these limitations, recent research has turned toward graph learning frameworks that infer network topology directly from data while enforcing sparsity and robustness. Among recent data-driven approaches, graph-learning frameworks aim to infer subject-specific network topology directly from the observed signals while enforcing sparsity and smoothness constraints. In this perspective, the network is learned under a smooth graph-signal assumption, which provides a principled mechanism to control sparsity and suppress spurious edges, consistent with Laplacian learning in smooth graph signal representations [18]. This approach differs conceptually from sparse connectivity estimation based on inverse covariance (precision) matrices, which relies on optimization schemes tailored to sparse inverse covariance estimation [19]. Graph-based modeling has also been increasingly adopted in neuroimaging to quantify structure–function discrepancies across cognitive decline [20].

Importantly, focusing on local (node-wise) graph properties rather than only global network summaries enables the detection of region-specific connectivity disruptions that may relate to the heterogeneous clinical manifestations of ASD. Building on this direction, the present study employs the Lean-NET framework to estimate subject-specific sparse functional networks from ABIDE data, thereby avoiding dense correlation matrices and ad hoc thresholding [21]. Local graph metrics (e.g., node degree, local efficiency, assortativity) are then computed at each node to capture localized alterations in network organization. To obtain statistically robust and interpretable region-level signatures, node-wise group differences are evaluated using Welch’s *t*-tests with false discovery rate (FDR) correction, explicitly addressing the multiple-comparison problem that can inflate false positives in high-dimensional connectomic analyses. The resulting top-ranked node signatures are subsequently evaluated using a linear SVM with leave-one-out cross-validation, linking classification performance to localized, statistically controlled network features. The central hypothesis is that Lean-NET–derived subject-specific networks reveal robust node-level alterations in ASD and that these localized signatures provide discriminative power for ASD–TD classification.

The main contributions of our work can be summarized as follows:

- A graph learning-based approach (Lean-NET) is employed to construct subject-specific functional brain networks.
- Analysis of localized graph-theoretical features to characterize region-specific connectivity alterations
- Statistical feature selection using Welch’s *t*-test with FDR correction
- Robust classification using linear SVM with leave-one-out cross-validation

**Figure 1:**
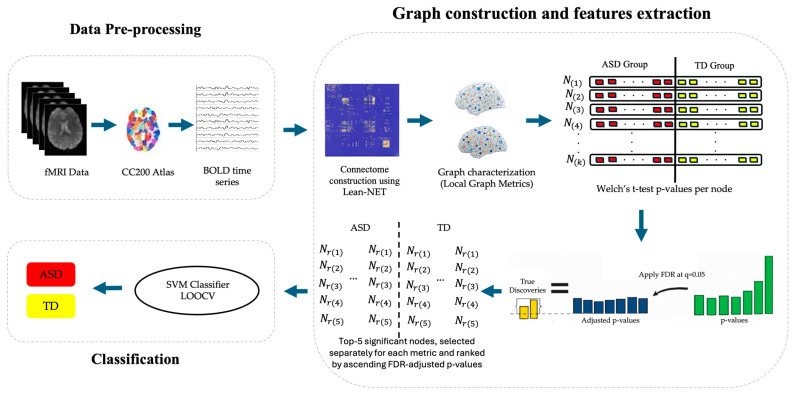
Proposed methodology for classification ASD using rs-fMRI BOLD signals, r(i) refers to the node ID with importance ranking i.

Through this methodology, shown in Figure 1, this approach aims to explore and visualize the FC patterns in the brain, providing insights into neural dynamics and differences between itself.

## 2. Methodology

### 2.1. Datasets Description

In this research, rs-fMRI data were obtained from the Autism Brain Imaging Data Exchange II [4], which was assembled from multiple independent neuroimaging sites, each approved by its respective local institutional review board. The dataset comprises rs-fMRI scans, consisting of 1112 participants including 539 individuals diagnosed with ASD and 573 TD. For the present analyses, only rs-fMRI data from the NYU site of the ABIDE II cohort were included. To ensure sufficient temporal sampling and improve the stability of subsequent functional connectivity and graph-based estimates, a minimum scan-length criterion was applied after preprocessing and quality control. Specifically, participants with recordings containing fewer than 140 rs-fMRI time points (T < 140) were excluded (5 ASD and 7 TD) because such short time series were considered insufficient for stable subject-level network construction [7]. After applying this criterion, the final analytical sample comprised 74 ASD and 98 TD participants, all satisfying T ≥ 140. Multi-site rs-fMRI acquisitions frequently differ in scanner hardware, acquisition protocols and image quality, thereby introducing site-specific variance that is unrelated to the actual neural differences between the groups. Restricting the sample to a single site, NYU provides a relatively large and methodologically homogeneous cohort, which is advantageous for developing and evaluating the proposed approach while minimizing site-related variability. The diagnostic status and demographic details of the participants were obtained from the ABIDE II database and are summarized in Table 1.

### 2.2. Data Preprocessing

Data were obtained from the New York University (NYU) site of the Autism Brain Imaging Data Exchange II (ABIDE II) dataset [4]. Resting-state fMRI was acquired using a gradient-echo echo-planar imaging sequence (TR = 2000 ms, TE = 15 ms, flip angle = 90°, voxel size = 3 × 3 × 4 mm^3^), with approximately 180 volumes per scan (≈6 min), whole-brain axial coverage, 4 mm slice thickness, and no interslice gap [4]. Preprocessing was performed using the Configurable Pipeline for the Analysis of Connectomes (C-PAC) and included standard steps such as slice-timing correction, motion realignment, spatial normalization to a standard template space, and nuisance signal regression [22]. Following preprocessing, regional BOLD time series were extracted using the Craddock 200 (CC200) functional atlas (200 ROIs) by computing the mean BOLD signal within each ROI, yielding region-wise time series for subsequent functional connectivity estimation and graph-based analyses [3]. Region-wise extraction and data handling were implemented using Nilearn [23].

### 2.3. Graph Construction and Features Extraction on BOLD Signals Using Lean-NET

#### 2.3.1. Connectome (Graph) Construction

The extraction of meaningful features from BOLD signals in fMRI is crucial for understanding brain function and pathology. In recent years, the graph-based network construction approaches and its extension, have emerged as powerful approaches for analyzing non-stationary and nonlinear signals such as BOLD signals. In this study, FC was not estimated using conventional pairwise correlations but via the novel approach Lean-NET framework, which yields a sparse FC representation specifically to overcome the limitations of fully connected networks for graph-theoretical analysis. In Lean-NET, sparsity is imposed through the solution of an optimization problem, rather than by standard thresholding methods as in standard fNETs. The thresholding method applies the same threshold to all subjects, whereas Lean-NET performs subject-specific sparsification.

For each subject, let $x\in R^{T\times N}$ denote the preprocessed resting-state BOLD time series A pairwise squared-distance matrix $Z\in R^{N\times N}$ is first computed as:

(1) $$Z_{ij}={∥x_{i}-x_{j}∥}_{2}^{2}$$

where $x_{i}\in R^{T}$ is the time series of ROI i. The functional connectivity graph $W\in R^{N\times N}$ is then learned using the Lean-NET implementation of the Kalofolias graph-learning model [24], which solves:

(2) $$\underset{W\geq 0}{min\left\langle W,Z\right\rangle }-\alpha \sum_{i}\log d_{i}+\beta ∥W\;{∥}_{F}^{2}$$

where $d_{i}=\sum_{j}W_{ij}\;$ denotes the degree of node i, and α=β=1. The resulting matrix is rescaled by a factor δ=2(W←δW) and symmetrized. To retain only robust positive connections, negative entries and edges with very small weights ($W_{ij}<0.03$) are set to zero.

The hyperparameters of the Lean-NET framework were selected based on prior literature rather than optimized for the present dataset. In particular, the regularization parameters were set to *α* = *β* = 1, following established practice in graph-learning formulations to balance data fidelity and sparsity constraints, as originally proposed by Kalofolias [24] and adopted in subsequent neuroimaging studies [18]. This setting provides a stable compromise between enforcing network sparsity and preserving meaningful functional relationships.

In addition, a small-weight pruning threshold ($W_{ij}<0.03$) was applied to remove residual weak connections and improve numerical stability, consistent with previous Lean-NET–based studies [18]. As these hyperparameters were fixed based on established literature, a preliminary sensitivity analysis was conducted to assess the robustness of this choice. The results indicated that the resulting network topology and the identification of discriminative nodes were not overly sensitive to these parameter settings. Specifically, the threshold primarily eliminated near-zero edges without affecting the dominant functional structure, confirming that the resulting sparse graphs were driven mainly by the Lean-NET optimization rather than by post hoc thresholding. This procedure yields subject specific adjacency matrices directly inferred from the ROI time series via the Lean-NET approach, which enforces smoothness and sparsity constraints to capture the underlying functional connectivity structure.

#### 2.3.2. Node Level Graph Metrics

After constructing the subject-specific functional networks using the Lean-NETmodel, we characterized each connectome through a set of node-level graph metrics to capture the local topological properties of brain regions. These measures quantify how each node contributes to information integration and segregation within the overall functional network.

For each subject, we computed the following metrics from the adjacency matrix *W* = [$w_{ij}$]; where $w_{ij}$ represents the connection weight between nodes *i* and *j*, the following local graph measures were computed for each subject:

1. **Local (Node) Assortativity (**$r_{i}$**)**: evaluates the tendency of nodes to connect with other nodes that have similar connectivity degrees, indicating the network’s local structural organization, the local assortativity of node *i* is defined as [25]:
  $$r_{i}=\frac{j\left(j+1\right)\left(\bar{k}-\mu_{q}\right)}{2M\sigma_{q}^{2}}$$
  where *j* is the node’s remaining degree, $\bar{k}$ represent the average remaining degree of the neighbors, and $\sigma_{q}\neq 0$. The $\mu_{q}$ is the global average excess degree.

2. **Betweenness centrality (**$B_{i}$**)**: is a measure of a node’s importance within the network, defined as the fraction of all shortest paths between any two other nodes in the network that pass-through node *i.* The shortest path lengths were calculated using the inverse of the weighted adjacency matrix *W*, as the edge lengths, and *B_i_* is computed using an algorithm tailored for weighted graphs as shown in Equation (3) [26].
  (3) $$B_{i}=\sum_{j\neq k\neq i}\frac{\;\sigma_{ij}\;(i)\;}{\sigma_{jk}}$$
  where $\sigma_{jk}$ is the total number of shortest paths between node *j* and node *k* and $\sigma_{jk\;}\left(i\right)\;$ is the number of those paths pass through *i*.

3. **Clustering coefficient (**$C_{i}$**):** The local clustering coefficient measures how tightly connected a node’s neighbors are to each other [26]. A commonly used method for calculating local clustering coefficients is in Equation (4):
  (4) $$C_{i=}\frac{2E_{i}}{K_{i}\left(K_{i}-1\right)}$$
  where the $E_{i}$ represents then number of edges for node *i*, and $K_{i}$ is the degree of node *i*.

4. **Local distance**: is a measure of the average shortest distance from node *i* to all other nodes in the network [16,26]. All are based on the shortest path length matrix $D^{g}$ where the length of an edge $D_{ij}^{g}\;$ is defined as the inverse of the weighted connection strength:
  (5) $$D_{ij}^{g}=\frac{1}{W_{ij}}$$

5. **Node Degree (**$K_{i}$**)**: is the most fundamental measure of nodal connectivity and is defined as the number of connections incident to node i, reflecting its level of topological integration within the network [26,27]. It is calculating using the binarized version of the adjacency matrix $A_{ij}$, where $A_{ij}=1$ if an edge between nodes i and j is present and $A_{ij}=0$ otherwise. For a graph *G* = (*V*, *E*) with N nodes, the degree $K_{i}$ of node *i* is given by
  (6) $$K_{i}=\sum_{j\in V}A_{ij}$$

6. **Local Efficiency (**$E_{i}$**)**: quantifies the efficiency of information exchange among the immediate neighbors of node *i*, representing the local fault tolerance of the network [16,26]. The Efficiency is defined as the inverse of the average shortest path length between all pairs of nodes in the subgraph:
  (7) $$E_{loc,i}=\frac{1}{K_{i\;\;}\left(k_{i}-1\right)}\sum_{j\in G_{i}}\sum_{h\in G_{i,h\neq j}}\frac{1}{d_{jh}}$$
  where $G_{i}$ is the set of neighbors on node i, $d_{jh}$ represents the shortest path length between the node *j* and *h* within the subgraph $G_{i}$ and $k_{i}$ is given in Equation (6). So the local $E_{i}$ is a key measure of functional segregation or modularity.

These metrics were calculated by brain connectivity toolbox (BCT) [26] for all brain regions to create feature vectors that describe each subject’s local connectivity pattern. Focusing on local rather than global properties help highlight region specific differences in how brain areas communicate, which is especially relevant since many studies report that ASD is related to localized hypo or hyper connectivity rather than broad, network-wide disruptions [28,29]. By capturing these nodal irregularities, the metrics provide a clearer picture of the connectivity patterns that may contribute to social and cognitive difficulties in ASD offering insights that global metrics, which only summarize overall network structure, cannot fully reveal [16,30].

#### 2.3.3. Statistical Testing and Leakage-Free Feature Selection

The first stage of the analysis focuses on statistical testing and feature selection to identify brain regions (nodes) that exhibit significant differences in local graph properties between ASD and TD groups. For each subject, node-wise local graph metrics were computed from the subject-specific functional network (e.g., node degree, local efficiency, assortativity, clustering coefficient, betweenness centrality, and local distance), capturing complementary aspects of local network organization.

To assess whether group differences in each metric were statistically significant, Welch’s two-sample *t*-test was applied independently at each node, yielding one *p*-value per node. Welch’s test was selected because it is robust to unequal variances and accommodates unequal group sizes, which are common in clinical neuroimaging datasets. To control for multiple comparisons across nodes, the resulting *p*-values were adjusted using the Benjamini–Hochberg false discovery rate (FDR) procedure at q=0.05 [31,32]. Nodes surviving FDR correction were considered statistically significant and were subsequently ranked in ascending order of their *p*-values to prioritize regions showing the strongest ASD–TD effects.

To evaluate the influence of feature dimensionality, incremental feature sets were formed by selecting the top-k ranked significant nodes for each metric (k=1–5). This procedure generated classifiers trained on progressively larger sets of discriminative node features, starting from the most significant node and extending to the top five nodes, thereby improving interpretability while limiting redundancy and over-parameterization [33].

#### 2.3.4. Classification

**Figure 2:**
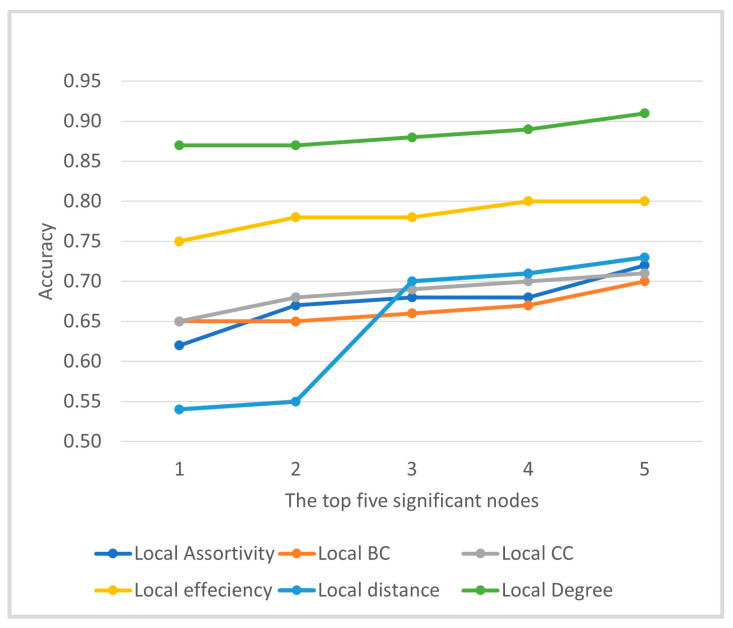
SVM classification accuracy using the top-ranked nodes (k=1,…,5) from each local graph metric.

To enhance model robustness and mitigate the curse of dimensionality associated with the 200-ROI feature space [34], classification was performed on low-dimensional feature sets defined by statistically prioritized nodes. For each local graph metric, nodes were ranked according to ASD–TD group differences based on Welch’s *t*-test *p*-values (with BH-FDR correction as described in Section 2.3.3), and separate models were trained using the top-k nodes. To examine the effect of feature dimensionality, this procedure was repeated for k=1–5, and the corresponding accuracy trends are reported in Figure 2.

The number of selected nodes k controls the dimensionality of the feature space used by the SVM. Very small k values may not capture sufficient discriminative information, whereas larger k increases model complexity and the risk of overfitting in a modest sample-size setting. As illustrated in Figure 2, performance improved within the low-dimensional range k=1–5, and k=5 was selected as a fixed operating point for Table 2 to provide a compact yet informative feature set and a consistent comparison across metrics.

ASD versus TD classification was conducted using a support vector machine (SVM) applied to node-wise local graph metrics derived from Lean-NET adjacency matrices. A linear-kernel SVM [35] was implemented in MATLAB_R2025b (fitcsvm). Predictor variables were z-score standardized, and class priors were set to uniform to mitigate class imbalance (74 ASD, 98 TD). Model performance was evaluated using leave-one-out cross-validation (LOOCV) [31], in which each subject served once as the test sample while the remaining subjects formed the training set. Classification accuracy was computed across LOOCV folds for each metric.

## 3. Results and Discussion

Among the local graph measures evaluated in Table 2, node degree yielded the highest overall classification performance, with accuracy, sensitivity, specificity, and F1-score approaching 0.90 under LOOCV. The local efficiency metric demonstrated intermediate yet relatively stable performance, characterized by high precision but lower sensitivity. In contrast, assortativity, betweenness centrality, clustering coefficient, and distance exhibited more limited discriminative capability, with classification accuracies ranging between 0.70 and 0.73.

As illustrated in Figure 2, the selection of nodes was limited to a range of k = 1 to 5 to avoid the curse of dimensionality, thereby focusing on the most informative and robust regions [36]. Among the local graph measures, node degree consistently yielded the highest classification performance across all values of k, followed by local efficiency, whereas metrics such as local assortativity, betweenness centrality, clustering coefficient, and distance exhibited comparatively lower accuracies. This pattern indicates that only a subset of local graph measures is highly sensitive to ASD-related differences and that focusing on the most discriminative nodes provides a compact yet interpretable representation of network alterations.

At the same time, the node-wise graph analysis demonstrated that ASD-related network differences were not uniformly distributed across the connectome but instead manifested a spatially selective pattern. Specifically, only a subset of regions showed statistically meaningful group differences, with local metrics like assortativity identifying approximately 30 discriminative nodes and degree identifying about 50 nodes. This pattern is consistent with previous findings that ASD-related changes are predominantly localized to key hubs within the default mode, salience, and subcortical networks, rather than spread across the whole brain (global alterations).

A more detailed inspection of the full set of statistically significant nodes further revealed that these spatially localized alterations collectively reflect a substantial global shift in functional network topology. Specifically, across the identified hub regions in the ASD group—particularly within frontal and temporal areas—a consistent increase in betweenness centrality and nodal distance was observed. This indicates a pattern of local hyperconnectivity in which these hubs assume a disproportionate role in local information flow while becoming increasingly segregated from distal network modules—a configuration consistent with the hypotheses of enhanced modular segregation and reduced long-range integration in ASD. Although concentrated in specific nodes, these changes are sufficient to modulate global efficiency by disrupting hub-mediated communication pathways. This provides a mechanistic bridge to established global theories of ASD, which emphasize a dual pattern of reduced long-range integration and increased local segregation. Our findings thus support the hypothesis that focal disruptions in hub organization can collectively manifest as widespread network-level dysfunction, a recognized hallmark of ASD neurobiology [16,37,38].

**Figure 3:**
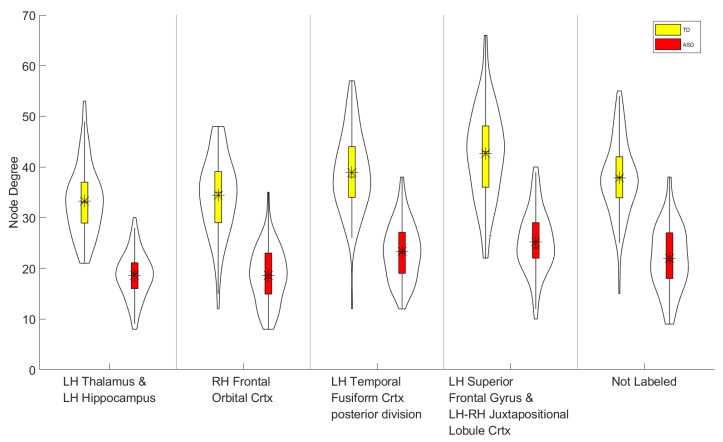
Violin plots showing the distribution of node degree values for the top five discriminative ROIs (selected from the degree metric by the smallest Welch’s *t*-test *p*-values after BH-FDR correction) in the ABIDE II–NYU cohort. TD participants are shown in yellow and ASD participants in red. For each ROI, the violin depicts the full distribution across subjects; the central marker indicates the mean, and the thick bar indicates the interquartile range. ROI labels are reported on the *x*-axis (LH/RH denote the left/right hemisphere; Crtx denotes cortex).

Given that the local degree metric yielded the highest classification performance, subsequent analyses focused on the node-degree distributions of key brain regions, as depicted in Figure 3. Each node is shown with its anatomical label and functional role. The thalamus, a major subcortical relay for sensory, motor, and attentional information, is frequently reported to exhibit atypical thalamo-cortical connectivity in ASD, which has been linked to sensory processing abnormalities and attentional difficulties [39,40]. The hippocampus, which is central to declarative memory, contextual integration, and social cognition, shows disrupted anterior–posterior specialization and altered coupling with cortical and subcortical regions, potentially reflecting impairments in social memory and large-scale network integration [41]. Frontal and orbitofrontal cortices, implicated in executive control, decision-making, and socio-emotional regulation, demonstrate both structural and functional connectivity alterations in ASD, in line with deficits in higher-order cognitive and social processes [42,43]. Temporal cortical regions, particularly posterior and superior divisions, are key for language, auditory processing, and social cognition; atypical connectivity between these areas and subcortical as well as other cortical nodes may contribute to core social-communication difficulties [44,45]. Nodes annotated as “not labeled” in the atlas, likely corresponding to transitional or boundary parcels, may index more diffuse network-level reorganization; however, such findings require cautious interpretation due to potential limitations of the parcellation scheme. Taken together, these observations indicate that ASD-related functional connectivity alterations are concentrated within subcortico-cortical networks supporting sensory processing, memory, executive function, and social cognition, rather than being uniformly distributed across the brain.

These findings indicate that the SVM classifier is sensitive to subtle alterations in brain network organization, and that graph-theoretical metrics indexing information integration and segregation are particularly informative for detecting ASD-related changes. Specifically, betweenness centrality is reduced in the ASD group, suggesting a diminished capacity of certain nodes to serve as critical intermediaries for information transfer across the network. Local efficiency is also lower in ASD, consistent with less effective integration of information within the immediate neighborhood of each node. In addition, reduced clustering coefficient together with increased distance in ASD reflects weaker local grouping of brain regions and less efficient communication pathways.

Several limitations should be considered when interpreting the present findings. First, the comparatively high classification performance relative to some ABIDE-based studies using correlation-derived functional connectivity is likely attributable to methodological choices—namely sparse, subject-specific network construction via Lean-NET and low-dimensional feature modeling—rather than increased model complexity; nevertheless, such gains may not generalize beyond the specific experimental setting. In particular, the exclusive reliance on single-site rs-fMRI data from the NYU cohort of ABIDE II constitutes a major limitation. Although single-site analysis reduces confounds related to scanner hardware, acquisition parameters, and site-specific variability, it substantially limits external validity. Inter-site heterogeneity is a well-recognized challenge in ABIDE datasets and can alter functional connectivity estimates, node-wise graph metrics, and downstream classification performance; consequently, discriminative node patterns and models learned under single-site conditions may not transfer reliably to other sites or clinical scenarios. Second, node-level graph measures are inherently parcellation-dependent, and the regional findings reported here should therefore be interpreted with respect to the CC200 atlas used in this study; alternative atlases or resolutions may yield different node rankings and effect local metrics. Importantly, while the proposed graph-learning and feature-selection pipeline is not intrinsically tied to a single atlas and can be extended to other parcellations, systematic evaluation across multiple atlases and explicit cross-site validation (e.g., training on one site and testing on another, or harmonized multisite protocols) are necessary to establish robustness and generalizability.

## 4. Conclusions

This study presents an exploratory, proof-of-concept framework for ASD–TD discrimination from rs-fMRI by combining subject-specific sparse functional network estimation (Lean-NET) with node-wise local graph metrics and an interpretable linear SVM. Using the ABIDE II NYU cohort, the proposed pipeline identified localized, statistically prioritized node-level signatures—most prominently degree-based features—that were associated with improved classification performance relative to several local metrics. These findings support the methodological premise that learning sparse, individualized graphs and focusing on local topology can yield compact and interpretable feature representations for characterizing ASD-related network differences.

Importantly, the current results should not be interpreted as evidence of near-clinical diagnostic applicability. The analyses were restricted to a single site and a single parcellation (CC200), and performance was evaluated under a limited-sample validation setting; therefore, generalizability to other acquisition sites, protocols, and populations remains unestablished. Future work should (i) validate the proposed framework in multi-site settings using appropriate harmonization and cross-site generalization tests, (ii) assess robustness across alternative atlases and resolutions, and (iii) relate discriminative node-level signatures to behavioral and clinical measures to strengthen neurobiological interpretability. Overall, the proposed approach provides a promising methodological basis for localized connectome modeling in ASD, while requiring broader external validation before any claims about diagnostic utility can be made.

## Figures and Tables

**Table 1:** Overview of analyzed subject’s demographics.

	**ASD (*n* = 74)**	**TD (*n* = 98)**	***p*** **_Value**
Age	14.7 ± 7.0	15.1 ± 6.0	0.678
Gender (Male/Female)	64/10	72/26	0.572
Full scale IQ	107.9 ± 16.6	113.2 ± 13.1	0.045

**Table 2:** Classification accuracies of linear SVM classifiers obtained via leave-one-out cross-validation using local graph metric separately across the 5 most statistically significant nodes for ASD vs. TD discrimination.

Metric	Accuracy	Sensitivity	Specificity	Precision	F1-Score
Assortivity	0.72	0.61	0.79	0.65	0.62
Betweenness centrality	0.70	0.68	0.80	0.70	0.64
Degree	0.91	0.91	0.91	0.90	0.89
Clustering Coefficient	0.71	0.67	0.74	0.66	0.67
Distance	0.73	0.74	0.72	0.67	0.70
Efficiency	0.80	0.68	0.80	0.83	0.75

## Data Availability

Publicly available datasets were analyzed in this study. Resting-state fMRI data were obtained from the Autism Brain Imaging Data Exchange II (ABIDE II) repository, NYU site. The dataset can be accessed upon registration from the ABIDE II website (http://fcon_1000.projects.nitrc.org/indi/abide/abide_II.html) (accessed on 20 June 2025). No new neuroimaging data were collected for this study.
